# Lithium-Induced Arginine Vasopressin Resistance (AVP-R): A Case of Chronic Exposure to Lithium

**DOI:** 10.7759/cureus.41677

**Published:** 2023-07-11

**Authors:** Andreia Lopes, Ana de Carmo Campos, Joana Marques Simões, Alda Jordão

**Affiliations:** 1 Clinical Pharmacology and Therapeutics Laboratory, Lisbon University Faculty of Medicine, Lisbon, PRT; 2 Internal Medicine, North Lisbon University Hospital Centre (CHULN) - Pulido Valente Hospital, Lisbon, PRT; 3 Department of Health Promotion and Prevention of Non-Communicable Disease, National Health Institute Doutor Ricardo Jorge, Lisbon, PRT; 4 Thorax Department and Pulmonology Service, North Lisbon University Hospital Centre (CHULN), Lisbon, PRT; 5 Internal Medicine, University of Lisbon Faculty of Medicine, Lisbon, PRT

**Keywords:** adh, antidiuretic hormone, neurologic toxicity, adverse drug reaction, avp-r, arginine vasopressin resistance, nephrogenic diabetes insipidus, lithium poisoning, lithium intoxication, lithium

## Abstract

Lithium salts (lithium) is a psychotropic drug widely used as a pharmacological option in managing bipolar disorder. Regular monitoring of serum levels is necessary due to the narrow therapeutic range of lithium. Typically, the diagnosis of lithium intoxication is based on the presence of elevated plasma levels. Nevertheless, poisoning can ensue from either acute ingestion or chronic use, even in patients with normal plasma levels. The utilization of lithium has been decreasing due to its potential for multiorgan toxicity. Lithium accumulation in renal distal tubular cells is a prevalent cause of acquired arginine vasopressin resistance (AVP-R), previously known as nephrogenic diabetes insipidus (DI). Some patients might also experience neurologic persistent symptoms after plasma level normalization, a condition known as the syndrome of irreversible lithium-effectuated neurotoxicity (SILENT). We present a case report of acquired AVP-R following prolonged lithium use. This case report aims to increase awareness, particularly among those who may be unfamiliar with the use of lithium and its associated adverse reactions. In addition, it seeks to highlight the dissociation between clinical manifestations and lithium plasma levels, emphasizing the need for careful evaluation in patients receiving lithium treatment.

## Introduction

Lithium is a psychotropic drug used as a first-line agent in the treatment of bipolar disorder, having a proven ability to reduce suicide attempts and suicidal death among bipolar patients [1]. Due to its narrow therapeutic index (0.6-1.2 mmol/L), close monitoring of serum levels is required [2]. Nonetheless, accidental or intentional ingestion and chronic use can potentially result in multiorgan toxicity, affecting various systems, such as renal, neurological, cardiac, gastrointestinal, thyroid, or parathyroid [3].

Lithium-induced nephrotoxicity englobes a variety of conditions, such as arginine vasopressin resistance (AVP-R), and, less frequently, chronic tubulointerstitial nephropathy, renal tubular acidosis, and hypercalcemia. The severity and form of presentation usually depend on the lithium dose, chronicity of exposure, volume status, and kidney function [4].

AVP-R, previously known as nephrogenic diabetes insipidus (DI), is characterized by the excretion of large volumes of dilute urine, due to an inability to concentrate urine in response to arginine vasopressin or antidiuretic hormone (ADH). Acquired forms of AVP-R may result from chronic lithium therapy, with an incidence ranging from 20% to 87% [4]. In these patients, AVP-R arises because of lithium accumulation in distal tubular cells [4]. Some studies suggest that lithium reduces aquaporin-2 (AQP2) gene transcription and impairs the expression of AQP2 channels in the principal cells of renal collecting tubules, leading to ADH resistance [5]. Others consider that lithium inhibition of signaling pathways involving glycogen synthase kinase 3 beta, may result in dysfunction of the AQP2 water channel, impairing urine concentration [6]. 

Some patients might also experience persistent neurologic symptoms after normalization of plasma lithium levels, a condition known as the syndrome of irreversible lithium-effectuated neurotoxicity (SILENT) [7].

The therapeutic approach to lithium toxicity involves discontinuation of lithium therapy. In patients with prolonged use of lithium, AVP-R and neurologic symptoms may persist despite discontinuation of the therapy/treatment [8]. Intravenous fluids are crucial to restore sodium and water balance in hypovolemic patients, helping to maximize lithium clearance. However, hemodialysis may be considered in patients with severe toxicity [9].

We present a case report of a patient with AVP-R and concomitant neurological symptoms, which can be attributed to chronic lithium exposure.

## Case presentation

A 61-year-old woman with bipolar disorder diagnosed 30 years ago, on treatment with lithium carbonate (1000 mg daily) for over two decades, presented to the emergency department (ED) with symptoms of lethargy, anorexia, muscle weakness, and psychomotor slowing. She has been undergoing regular psychiatric surveillance and diligent monitoring in place. One week prior to the current presentation, she had initiated lamotrigine (100 mg twice daily) and bupropion (150 mg once daily) as a part of her treatment plan, in response to the progressive deterioration of her depressive phase.

Initial assessment revealed normal vitals (blood pressure 121/62 mmHg, pulse rate 72 bpm, respiratory rate 23 bpm, and adequate oxygen saturation in room air SpO_2_>97%) and apyrexia. The patient appeared dehydrated. Despite being oriented and cooperative, she exhibited signs of slowed mentation, easy distractibility, alogia, and prompted and impoverished speech. No focal neurological deficits were observed; however, she displayed signs of appendicular ataxia and generalized muscle weakness (with a motor strength of 3/5 based on the Medical Research Council (MRC) scale).

Laboratory findings revealed leukocytosis (29.9x10^9^/L), acute kidney injury (Acute Kidney Injury Network (AKIN) II), metabolic acidosis (pH 7.406, pCO_2_ 31.9 mmHg, HCO_3_ 21.4 mmol/L, lactate 24 mmol/L), mild hyperlacticaemia and hyperkaliemia and hypernatremia (sodium (Na) 164 mmol/L) (Table 1). No elevation in acute-phase parameters was observed during the assessment (C-reactive protein (CRP) 0.2 mg/dL; procalcitonin 0.15 ng/mL) (Table 1).

**Table 1:** Main laboratory parameters. AST: aspartate aminotransferase; ALT: alanine aminotransferase; GGT: gamma-glutmyl transferase; pCO2: partial pressure of carbon dioxide; pO2:  partial pressure of oxygen; TSH: thyroid-stimulating hormone; T4: thyroxine

Parameter	Value	Reference range
Hemoglobin	14.7 mg/dL	12.0–15.3 mg/dL
Hematocrit	42.9%	40–50%
Mean corpuscular volume	95 fL	80–97 fL
Leukocytes	29.9x10^9^/L	4.0–11.0x10^9^/L
Neutrophils	15.0x10^9^/L	1.9–7.5x10^9^/L
Monocytes	1.4x10^9^/L	0.1–1.0x10^9^/L
Platelets	408x10^9^/L	150–450x10^9^/L
C-reactive protein	0.2 mg/dL	<0.5 mg/dL
Procalcitonin	0.15 ng/mL	<0.5 ng/mL
AST	34 U/L	0–40 U/L
ALT	20 U/L	0–41 U/L
GGT	209 U/L	0–60 U/L
Total bilirrubin	0.42 mg/dL	<1.2 mg/dL
Amylase	75 U/L	40–140 U/L
Total creatine kinase	13 U/L	39–308 U/L
TSH	1.85 uU/mL	0.5–5.0 uU/mL
Free T4	1.12 ng/dL	0.8–1.8 ng/dL
D-dimer	0.25 mg/mL	0.0–0.5 mg/mL
Glucose	111 mg/mL	70–110 mg/dL
Creatinine	2.62 mg/mL	0.5–0.9 mg/dL
Urea	131 mg/mL	16–49 mg/dL
pH	7.406	7.35–7.45
pCO_2_	31.9 mmHg	35–45 mmHg
pO_2_	63.0 mmHg	75–100 mmHg
Bicarbonate	21.4 mmol/L	22–26 mmol/L
Lactate	2.4 mmol/L	0.4–2.0 mmol/L
Sodium	164 mmol/L	135–145 mmol/L
Potassium	5.6 mmol/L	3.5–5.1 mmol/L
Osmolality	335 mOsmol/kg	275–295 mOsmol/kg
Vasopressin	2.0 pg/mL	1–5 pg/mL
Copeptin	43.91 pmol/L	2.4–28.2 pmol/L
Urinary sodium	42 mmol/L	54–190 mmol/L
Urinary potassium	21 mmol/L	20–80 mmol/L
Urinary chloride	35 mmol/L	46–168 mmol/L
Urine osmolality	248 Osmol/Kg	300–900 mOsmol/Kg

Thoracic X-ray and electrocardiogram showed no relevant findings. Brain computed tomography (CT) showed no evidence of acute vascular lesions or space-occupying mass. The serum lithium level was found to be supratherapeutic at 2.67 mmol/L (reference range 0.6-1.2 mmol/L), prompting the initiation of intensive intravenous (IV) fluids (normal saline solution, 0.9% NaCl, 3 L/24 hours), and diuretics (furosemide IV, 20 mg 8/8 hours) at the ED (did not meet urgent dialysis criteria). Accidental or intentional lithium overdose was ruled out. These findings suggested lithium poisoning secondary to chronic use, causing neurological and renal dysfunction. The patient was hospitalized for treatment and therapeutic adjustment.

**Figure 1:**
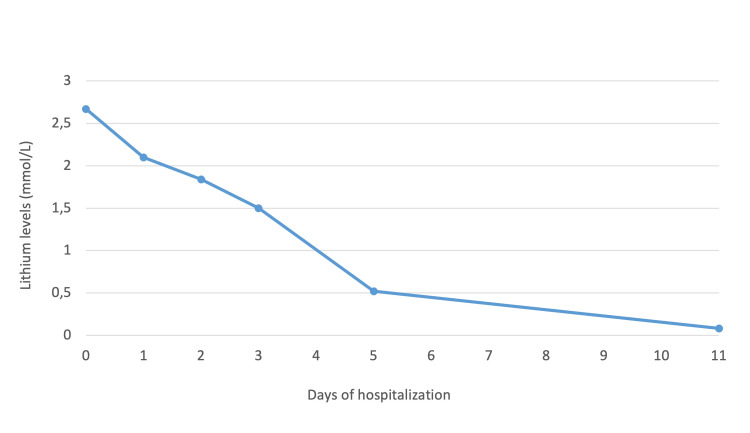
Lithium serum levels during hospitalization. Lithium serum levels reference range: 0.6–1.2 mmol/L

Lithium was discontinued upon admission, and on the second day of hospitalization, therapy with alternative psychotropic medications was initiated (sodium valproate, quetiapine, and haloperidol). Throughout the hospitalization period, serial plasma lithium levels returned to normal on the fourth day following the discontinuation of lithium, gradually decreasing to subtherapeutic levels by the 11th day (0.08 mmol/L) (Figure 1). However, the patient remained lethargic, with muscle weakness and psychomotor slowing. A second brain CT scan was conducted, revealing no notable changes compared to the previous scan.

Additional investigations revealed a severe hypernatremic hyperosmolar state (Na 164 mmol/L, reference range 135-145 mmol/L; osmolality (Osm) 335 mOsmol/kg, reference range 275-295 mOsmol/kg) (Table 1). Urinary electrolytes were ordered showing a hypotonic polyuria (urinary Na (Na_U)_ 42 mmol/L, reference range 54-190 mmol/L; urinary K (K_U_) 21 mmol/L, reference range 20-80 mmol/L; urinary chloride (Cl_U_) 35 mmol/L, reference range 46-168 mmol/L; urine osmolality (Osm_U_) 248 mOsmol/Kg, reference range 300-900 mOsmol/Kg) (Table 1), with a urine output of more than 50 mL/kg/24 hours (maximum of 2950 mL/day during the hospitalization). Vasopressin levels were within the normal reference range (Table 1), and the urine concentration capacity was found to be less than 50% (35.1%) following the administration of desmopressin (Osm_U_ 387 mOsmol/kg). The copeptin level was 43.91 pmol/L (reference range 2.4-28.2 pmol/L).

AVP-R (or nephrogenic DI) was considered as a likely result of chronic lithium intoxication. Hypernatremia corrected after five days of enteric administration of free water (Na 145 mmol/L and Osm 292 mOsmol/kg). At the time of discharge on the 13th day (D13), the patient exhibited normal serum sodium levels of 145 mmol/L and osmolality of 292 mOsmol/kg.

Despite no signs of irreversible neurological dysfunction, the patient showed a very slow improvement in ataxia and muscle weakness. During the hospitalization, the motor rehabilitation plan was intensified, leading to a favorable response from the patient. Upon discharge, the patient was transferred to a rehabilitation center, exhibiting progressive improvement in muscle weakness (MRC grade 4+/5) and minimal residual appendicular ataxia. Her speech remained coherent, and her mood remained stable. The patient demonstrated progressive autonomy and was able to walk independently under supervision.

During a follow-up appointment one week after discharge, the patient exhibited normal sodium levels and preserved urinary concentrating ability. In addition, there was minimal residual appendicular ataxia and muscle weakness in the lower limbs (MRC 4+/5). An electromyography (EMG) was performed, revealing a neurogenic lesion on the L5 root with signs of denervation, in relation with compression. This finding helped to rule out lithium-induced irreversible neurotoxicity as the cause of the persistent muscle weakness in the lower limbs. Since hospitalization, the patient has remained on treatment with psychotropic medications other than lithium, with a good outcome.

## Discussion

AVP-R results from a reduced sensitivity to antidiuretic effect of AVP when in physiological levels. While some cases are related to mutation of the AQP2 water channel and AVPR2 gene, most cases of AVP-R are acquired, including lithium exposure, hypercalcemia, hypokalemia, infiltrating lesions, urethral obstruction, and vascular diseases [10]. Lithium is recognized as one of the leading causes of acquired AVP-R, with reported prevalence ranging from 20% to 87% involving patients undergoing lithium treatment [4].

Lithium is a monovalent cation that is widely regarded as the drug of choice for the treatment of bipolar disorder. However, its pharmacologic mechanism is not established [11]. Lithium is efficiently and rapidly absorbed through oral administration, with a bioavailability close to 100%. It has the potential to accumulate in various organs, such as the liver, brain, and kidneys. The metabolism of lithium is minimal, with nearly 95% of the drug being excreted by the kidneys. The elimination half-life can range from 12 to 27 hours, but it may extend to 48 hours in individuals with chronic exposure and up to 58 hours in elderly patients due to impaired renal function [12]. Therefore, acute or chronic kidney disease may increase the risk for lithium accumulation and toxicity [4].

Due to its narrow therapeutic index, individuals undergoing lithium treatment should have their lithium plasmatic levels monitored to ensure efficacy and to avoid adverse events [2]. Lithium plasmatic levels over 2 mmol/L are considered toxic. However, patients chronically on lithium, who show significantly elevated concentrations, might be asymptomatic, as lithium exerts its effects in the intracellular compartment and not in the intravascular space. As a result, there is a limited correlation between the clinical manifestations of lithium toxicity and plasma levels of the drug [13].

Lithium intoxication may be classified as acute, acute on chronic, or chronic, depending on the exposure profile. The first two types result from excessive intake, including suicidal intent, accidental ingestion, or dose modifications in chronically treated patients. Patients who receive chronic lithium treatment are at an increased risk of lithium poisoning, which can occur due to various factors that impair lithium elimination. These factors include conditions, such as sodium depletion and dehydration, as well as the use of nephrotoxic drugs that can reduce the glomerular filtration rate [8]. Hence, lithium can initiate a detrimental cycle of toxicity whereby AVP-R-induced polyuria exacerbates hypovolemia, impairs renal function, reduces lithium excretion, increases plasma concentration of the drug, and further compromises the kidney's ability to concentrate urine [4].

Patients with lithium poisoning may present with nausea, vomiting, diarrhea, and abdominal pain when in acute exposition. If there is no impairment in the kidney function, neurotoxicity is unlikely to develop. Chronic lithium toxicity is characterized by a progressive accumulation of lithium in the kidneys or central nervous system, especially in individuals who do not drink enough water. This process contributes to the development of neurotoxic effects associated with long-term lithium exposure, such as sluggishness, ataxia, confusion, agitation, and neuromuscular excitability. Severe toxicity may present as seizures, encephalopathy, and death [8].

Normally, the resulting thirst and increased intake of free water compensate all the fluid losses and avoid hypernatremia. However, it should be noted that patients on depressive phase of bipolar disorder may have reduced self-water intake, aggravating the dehydration status related to lithium-induced AVP-R and impairing hypernatremia correction. In this situation, fluid replacement may represent a cornerstone of the AVP-R treatment. Our patient's dehydration was a consequence of her refusal to consume food and liquids in the days preceding their hospitalization. The cycle of toxicity resulting from chronic lithium use exacerbated AVP-R, leading to further dehydration, renal damage, and lithium accumulation. In this case, we implemented a carefully controlled and gradual fluid infusion to compensate for the patient's decreased water intake. This approach was necessary to prevent sudden compensation for hypernatremia, which could potentially elevate the risk of cerebral edema.

Management goals of lithium intoxication aim to prevent long-term and irreversible neurotoxicity and to ensure the patient's survival. Lithium should be discontinued unless benefits outweigh the risks [8]. As no antidote is available, the treatment approach includes fluid replacement to correct hypovolemia and promote lithium excretion. In cases of severe toxicity (level of 2.0 mmol/L and above), hemodialysis should be considered, irrespective to lithium concentration. In patients showing plasmatic levels above 4 mmol/L and concomitant severe kidney function impairment, some studies showed the benefits of hemodialysis. For all others, diuretics seems to be sufficient as supportive care [14]. Our patient did not meet the dialysis criteria, as lithium concentration on admission was 2.67 mmol/L and she did not present severe neurologic toxicity. Thus, she started free water intake and diuretic therapy, which was maintained until water deficit and electrolyte imbalance complete correction. Lithium concentrations were measured once daily to manage treatment efficacy, until reaching no detected level.

Lithium-induced AVP-R is typically self-limiting and does not pose significant clinical danger. However, in cases of chronic use, lithium intoxication may lead to irreversible neurotoxicity [8]. Patients with moderate to severe forms of lithium intoxication usually present polyuria, polydipsia, and nocturia [4]. Lab studies show hypertonic hypernatremia, dehydration, lactic acidosis, and acute renal lesion, as illustrated in this case. Complications of volume depletion include hypotension; vital organ hypoperfusion, namely, renal dysfunction with consequent acute tubular necrosis; and shock. Moreover, neurologic manifestations may occur due to osmotic water shifts as dehydration imposes, including irritability, cognitive impairment, altered mental status, seizures, cerebral infarction, and death [10]. In our patient, the observed neurological symptoms may be attributed to both the elevated plasma concentration of lithium and the severe hypernatremia resulting from AVP-R.

In most patients with AVP-R, low-sodium and low-protein diet, adequate hydration, replacement of water deficit, and diuretic therapy may be combined to correct hypernatremia and restore an euvolemic status. If oral fluid intake is not suitable, namely, due to impaired thirst mechanism, IV hypotonic fluids should be initiated. Diuretic therapy, specifically thiazides, plays a role in the treatment of lithium-induced AVP-R. Thiazides exert their effect by promoting sodium and water reabsorption in the proximal tubule through the activation of the renin-angiotensin-aldosterone system. This mechanism ultimately helps to reduce urine output and improve water reabsorption in patients with AVP-R. Amiloride is also an option, as it decreases transcellular lithium transport, blocking the effect of lithium on the AQP2 channel expression. The combination of thiazides and amiloride should be considered, as it reduces the risk of hypokalemia and metabolic alkalosis induced by thiazides [15].

The prevalence of lithium-induced AVP-R has decreased due to the declining utilization of lithium therapy. While it remains a prevalent adverse effect among patients receiving lithium, the overall incidence of lithium-induced AVP-R has become relatively rare [16].

In this patient, a diagnosis of lithium-induced AVP-R was established based on several factors. These included the patient's history of chronic lithium use; laboratory values, such as serum and urinary osmolality and electrolytes; and lack of response to desmopressin administration. Moreover, there was an exclusion of i) osmotic diuresis, ii) potential causes of central DI; such as tumors or trauma affecting the hypothalamus or pituitary gland, infiltrative diseases, hypoxic/ischemic encephalopathy, and neurosurgery with severe damage of the hypothalamus; and iii) primary polydipsia, which may also be caused by lithium intoxication. Our patient presented with hyperosmolar hypernatremia with low urine osmolality, making primary polydipsia unlikely, as these patients will be hyponatremic or normonatremic. Moreover, individuals with osmotic diuresis show hypernatremia along with elevated urine osmolality, usually over 600 mOsmol/Kg. Therefore, DI emerged as the most probable hypothesis, which was subsequently confirmed by the response to the desmopressin test, suggesting AVP-R. Furthermore, the presence of high copeptin levels (>21.4 pmol/L), which serves as a biomarker for the differential diagnosis of polyuria polydipsia syndrome, provides additional support for the AVP-R hypothesis [17].

After discontinuation of lithium and normalization of serum levels, she also revealed persistent signs of appendicular ataxia and generalized muscle weakness, which arose suspicions on SILENT. SILENT is characterized by neurologic and neuropsychiatric symptoms that develop along with an elevated lithium concentration and persist despite successful removal of the drug. Cerebellar dysfunction, brain stem dysfunction, extrapyramidal symptoms, and dementia are the most common sequels. The mechanism remains unclear, but it is hypothesized that it may result from demyelination caused by lithium at multiple sites in the nervous system [7]. There are many risk factors for developing lithium-induced neurologic sequelae, such as fever, acute intoxications or abrupt discontinuation of lithium, rapid correction of hypernatremia or hyperlithemia, concomitant use of antidepressants and antipsychotics, neurologic illness, hypertension, and chronic renal disease. In this way, it is essential to continuously monitor lithium serum levels and establish stricker exclusion criteria for initiating therapy [18]. To further investigate and exclude SILENT, an EMG was also performed, which provided evidence ruling out lithium-induced irreversible neurotoxicity.

Given that our patient initiated new antidepressant medications in the week prior to admission, we also assessed potential pharmacological interactions that could explain the observed toxicity. Neither lamotrigine nor bupropion interacts with lithium. However, it is important to note that patients taking thiazide diuretics are at higher risk of lithium poisoning, as these diuretics can reduce the renal excretion of lithium. This highlights the importance of lithium concentration monitoring while on thiazide for AVP-R treatment. Following the discontinuation of lithium therapy, alternative psychotropic medications were initiated to maintain the stabilization of bipolar disorder in the patient.

As the pharmacogenomics field is arising, some studies suggest that the variation in lithium response among individuals may be genetically determined. A recent systematic review found multiple studies on lithium pharmacogenetics showing a correlation between genetic variations and responses to lithium in bipolar disease, with a high number of genes involved [19]. However, due to methodological limitations, conclusive recommendations cannot be established. Moreover, responses to lithium seem to be polygenic, or when single genes are involved, it may have multiple polymorphisms. Therefore, no specific actionable clinical measures are currently recommended in the existing pharmacogenomics guidelines [19].

## Conclusions

Lithium remains the most effective treatment for bipolar disorder. However, its utilization has been declining due to the potential risks of long-term use, including the induction of multiorgan toxicity. Patients on chronic therapy should undergo plasmatic drug measurements on a regular basis to detect toxic levels, which increases the likelihood of nephrotoxicity and neurotoxicity. However, clinical monitoring is of uttermost importance, as patients can present toxicity-related symptoms even with normal lithium plasmatic levels. The implementation of protocols for the management of lithium intoxication, both in acute and chronic cases, is of significant importance. Some of the most important measures include avoiding dehydration, which leads to worsening of symptoms and increased toxicity, and initiating fluid replacement, particularly in those with impaired oral fluid intake and consequent hypernatremia. 

It is essential to report any adverse reactions that may arise from long-term lithium use and to remain vigilant regarding potential drug interactions involving this medication. Further studies are needed to expand the evidence and the potential role of pharmacogenomics in lithium prescription. This evidence would contribute to the development of comprehensive guidelines, encompassing actionable clinical measures and ultimately prioritizing patient safety.
